# Early port site and peritoneal metastasis following robot-assisted radical cystectomy: a rare case report

**DOI:** 10.1007/s00432-023-05562-9

**Published:** 2024-01-25

**Authors:** Shashank Tripathi, Deepak Prakash Bhirud, Mahendra Singh, Vikram Singh, Gautam Ram Choudhary, Shivcharan Navriya, Rahul Jena, Arjun Singh Sandhu

**Affiliations:** Department of Urology, All India Institute of Medical Sciences, Jodhpur, Rajasthan India

**Keywords:** Bladder cancer, Peritoneal metastasis, Port-site metastasis, Robot-assisted radical cystectomy, Rare

## Abstract

Radical cystectomy with pelvic lymph node dissection is the recommended treatment for managing muscle-invasive carcinoma of the urinary bladder. Early recurrence is observed in only about 4.1% of cases. Port-site metastasis following robot-assisted radical cystectomy is extremely rare. We encountered a challenging and a rare case of bladder cancer that manifested with port-site and peritoneal metastasis within 6 weeks of surgery.

## Introduction

Radical cystectomy with pelvic lymph node dissection is the recommended treatment for managing muscle-invasive carcinoma of the urinary bladder (Flaig 2019). Muscle-invasive disease is associated with frequent recurrences, with over 80% of recurrences occurring within the first 2 years after surgery (Nieuwenhuijzen et al. 2014). Early recurrence is observed in only about 4.1% of cases (Collins et al. 2017). Recently, robot-assisted radical cystectomy (RARC) has shown promise in delivering similar results with the added benefits of minimally invasive surgery. Port-site metastasis following RARC is rare, with only 11 reported cases in the literature (Port-site 2022). There are specific intraoperative factors, including traumatic tissue removal, tumor morcellation, excessive organ handling, spillage of urine, excessive pneumoperitoneum and the absence of bag retrieval, which have been associated with a heightened risk of local tumor recurrence in minimally invasive surgeries (Acar et al. 2014). Here, we present a challenging case of bladder tumour that manifested with port-site and peritoneal metastasis within 6 weeks of surgery.

## Case description

**Fig. 1:**
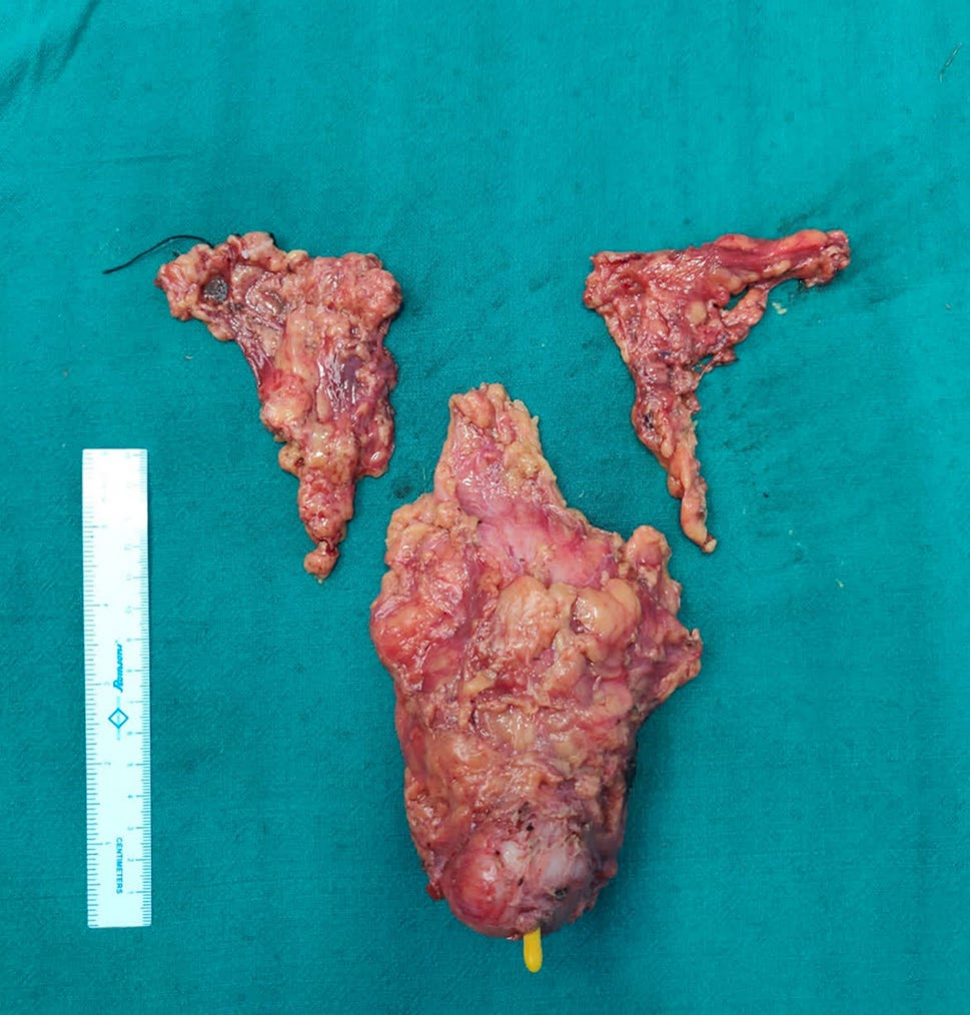
**a** Forceps showing the mesenteric metastasis and **b** CT image showing cutaneous port site metastasis (white arrow)

**Fig. 2:**
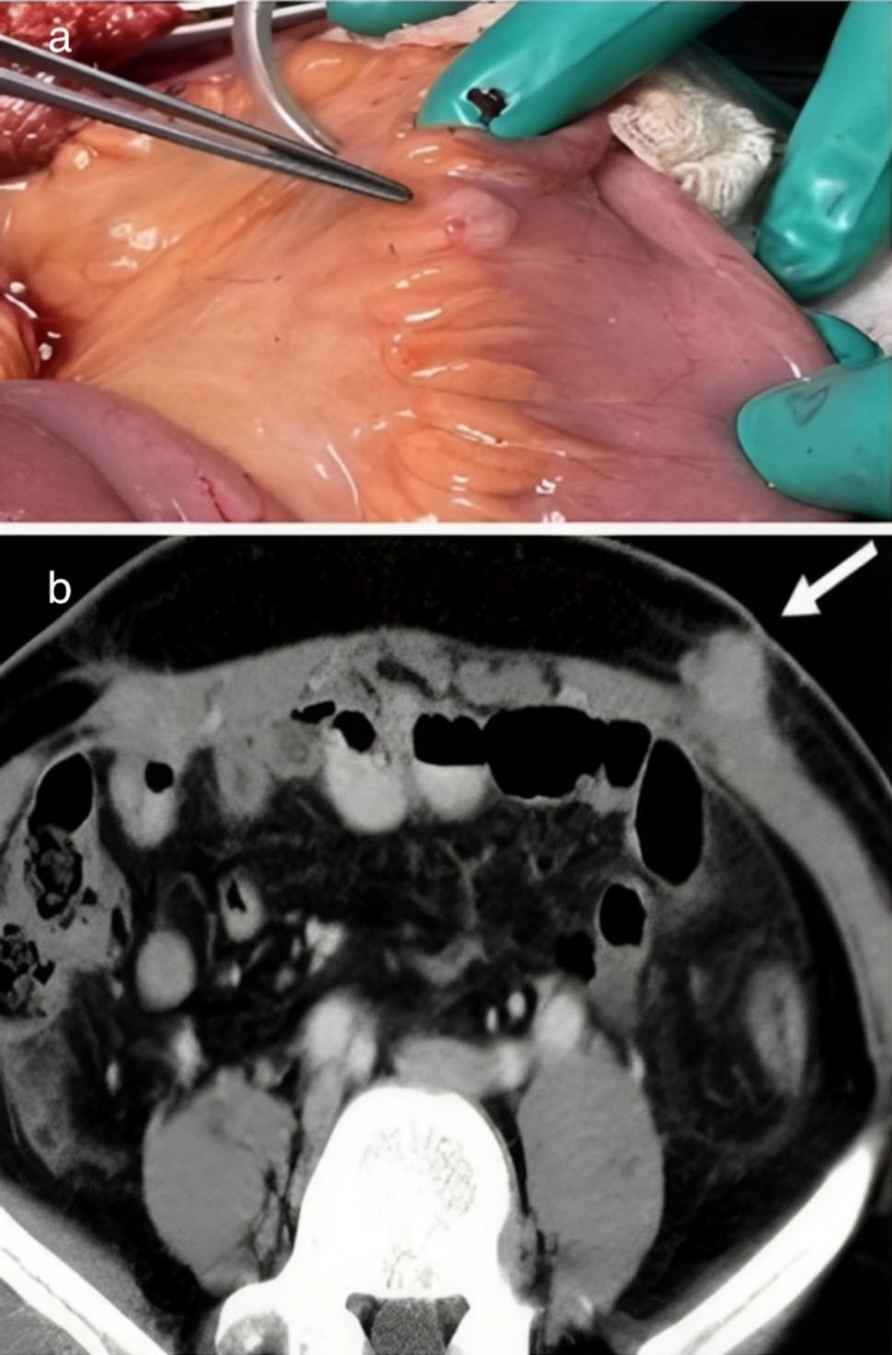
Specimen of radical cystectomy with bilateral pelvic lymph nodes, showing no evidence of any compromise or breach

A 44-year male patient without any comorbidities, presented with complaints of haematuria and dysuria. CT urography revealed a 3 × 3 cm urinary bladder mass at left posterolateral wall. Subsequently, he underwent transurethral resection of bladder tumour. The histopathology (HPE) report confirmed a diagnosis of high-grade muscle-invasive carcinoma (T2). PET scan showed no metastasis except for single left iliac lymph nodes involvement (SUVmax ~ 3.3). The treatment options, including neoadjuvant chemotherapy, were discussed with the patient. Despite counselling, the patient was reluctant to undergo chemotherapy and requested early surgery. After providing ample time for the decision, the patient persisted with the choice of direct surgery, understanding the associated morbidity and mortality risks. He then underwent RARC with bilateral extended pelvic lymph node dissection and extracorporeal ileal conduit diversion. The specimen was removed using an endobag, and the urinary diversion was performed extracorporeally through a lower midline infraumbilical incision of approximately 5 cm. This incision specifically facilitated the extraction of the specimen contained within the endobag (Fig. 1). There was not any intraoperative spillage of the tumour. The HPE confirmed high-grade invasive urothelial carcinoma (pT2N2Mx) with two out of 40 lymph nodes showing positive involvement, including evidence of extranodal tumour extension. Urethral and ureteral margins were negative for malignancy. Postoperatively, the patient developed catheter-associated urinary tract infection, fever, and later, upper respiratory tract infection, which precluded adjuvant chemotherapy. Furthermore, within 4 weeks of surgery, the patient complained of decreased urine output and deranged creatinine levels. Subsequent imaging revealed a suspicious pelvic lesion and evidence of bilateral pyelonephritis on a CT scan, with cutaneous metastasis at the left subcostal previous port site (Fig. 2b).

The patient underwent bilateral percutaneous nephrostomy placement, which led to gradual improvement in deranged creatinine levels. However, after 7 days, he developed abdominal distention and obstipation, requiring exploratory laparotomy and ileostomy formation. Intraoperatively, we observed multiple tumour deposits in the omentum, mesentery, and peritoneum (Fig. 2a). In addition, a frozen pelvic mass was compressing the small bowel and ileal conduit, making pelvic dissection and bowel mobilization challenging. As a result, we decided to perform diversion ileostomy. Port site metastasis was also excised and sent for biopsy separately. HPE confirmed the metastasis at port sites, mesenteric and peritoneal deposits. In post-op period, patient had severe malnutrition and weight loss.

Despite intensive medical care, his condition could not be improved, and he expired 25 days after the second surgery.

## Discussion

Open radical cystectomy has been associated with a recurrence probability of 5–10% in the pelvis and approximately 50% over a 5-year period (Ghoneim et al. 2008). Most recurrences occur within the first 2-year post-surgery. However, metastasis within 1 month of surgery is exceptionally rare, with only 11 cases reported in the medical literature (Port-site 2022). Common sites of metastasis include lymph nodes, lungs, liver, and bone (Shinagare et al. 2011). Atypical metastases such as peritoneal carcinomatosis, port-site metastasis, and extra-pelvic lymph node metastasis have been described (Nguyen et al. 2015). In our case, there was no evidence of tumour margin violation, and lymph node dissection was thorough (with only 2 out of 40 lymph nodes being positive with extranodal extension). Therefore, we believe that microscopic tumour deposits may have contributed to such early and aggressive metastasis.

Various theories have emerged concerning intraoperative techniques and their potential role in the development of peritoneal and port-site metastases. Among these theories are the ‘seed and soil’ hypothesis, which suggests that turbulent airflow during the establishment of pneumoperitoneum around the port site could lead to the implantation of tumor cells. This phenomenon, often referred to as the ‘chimney effect,’ is one of the proposed mechanisms (Langley and Fidler 2011). Concerns have also surfaced regarding peritoneal desiccation and compromised immune responses due to the use of dry CO2 for insufflation, prompting several in-vitro and animal studies to explore these emerging issues.

Unfortunately, the patient could not receive adjuvant chemotherapy due to respiratory and urinary tract infections and subsequent sepsis in quick succession. The patient hailed from a remote rural area and was unwilling to undergo frequent follow-up for neoadjuvant chemotherapy. Such cases underscore a practical challenge faced by surgeons in India, where many patients are non-compliant with treatment and present at advanced stages of the disease. Given the aggressive nature of the disease, neoadjuvant chemotherapy might have been a more suitable course of action.

## Conclusion

Early recurrence and atypical metastases, as observed in this case, serve as reminders of the need for comprehensive preoperative counselling, patient education, and shared decision-making. It also emphasizes the potential benefits of neoadjuvant chemotherapy in cases with aggressive disease characteristics, which could help improve outcomes in similar clinical scenarios.

## Data Availability

The data used in the article are present within the manuscript only.

## References

[CR1] Acar O, Esen T, Bavbek S et al (2014) Port site and peritoneal metastases after robot-assisted radical prostatectomy. Int J Surg Case Rep 5:131–134. 10.1016/j.ijscr.2014.01.010 24531016 10.1016/j.ijscr.2014.01.010 PMC3955239

[CR2] Collins JW, Hosseini A, Adding C, Nyberg T, Koupparis A, Rowe E et al (2017) Early recurrence patterns following totally intracorporeal robot-assisted radical cystectomy: results from the EAU Robotic Urology Section (ERUS) Scientific Working Group. Eur Urol 71(5):723–726 27816299 10.1016/j.eururo.2016.10.030

[CR3] Flaig TW (2019) NCCN guidelines updates: management of muscle-invasive bladder cancer. J Natl Compr Canc Netw. 17(5.5):591–3 31117036 10.6004/jnccn.2019.5017

[CR4] Ghoneim MA, Abdel-Latif M, El-Mekresh M, Abol-Enein H, Mosbah A, Ashamallah A et al (2008) Radical Cystectomy for carcinoma of the bladder: 2,720 consecutive cases 5 years later. J Urol 180(1):121–127 18485392 10.1016/j.juro.2008.03.024

[CR5] Langley RR, Fidler IJ (2011) The seed and soil hypothesis revisited—the role of tumor-stroma interactions in metastasis to different organs. Int J Cancer 128:2527–2535. 10.1002/ijc.26031 21365651 10.1002/ijc.26031 PMC3075088

[CR6] Nguyen DP, Al Hussein Al Awamlh B, Wu X, O’Malley P, Inoyatov IM, Ayangbesan A et al (2015) Recurrence patterns after open and robot-assisted radical cystectomy for bladder cancer. Eur Urol. 68(3):399–405 25709026 10.1016/j.eururo.2015.02.003 PMC4727829

[CR7] Nieuwenhuijzen JA, de Vries RR, van Tinteren H, Bex A, Van der Poel HG, Meinhardt W et al (2014) Follow-up after cystectomy: regularly scheduled, risk adjusted, or symptom guided?: Patterns of recurrence, relapse presentation, and survival after cystectomy. Eur J Surg Oncol EJSO 40(12):1677–1685 24462548 10.1016/j.ejso.2013.12.017

[CR8] Port-site metastasis and atypical recurrences after robotic-assisted radical cystectomy (RARC): an updated comprehensive and systematic review of current evidences—PubMed [Internet]. [Cited 2022 Jan 18]. Available from: https://pubmed.ncbi.nlm.nih.gov/32152900/10.1007/s11701-020-01062-x 32152900

[CR9] Shinagare AB, Ramaiya NH, Jagannathan JP, Fennessy FM, Taplin M-E, Van den Abbeele AD (2011) Metastatic pattern of bladder cancer: correlation with the characteristics of the primary tumor. Am J Roentgenol 196(1):117–122 21178055 10.2214/AJR.10.5036

